# Sexual difference of small RNA expression in Tetralogy of Fallot

**DOI:** 10.1038/s41598-018-31243-6

**Published:** 2018-08-27

**Authors:** Bo Wang, Guocheng Shi, Zhongqun Zhu, Huiwen Chen, Qihua Fu

**Affiliations:** 1 Department of Laboratory Medicine, Shanghai Children’s Medical Center, Shanghai Jiao Tong University School of Medicine, Shanghai, 200127 P.R. China; 2 Department of Cardiothoracic Surgery, Heart Center, Shanghai Children’s Medical Center, Shanghai Jiaotong University School of Medicine, Shanghai, 200127 P.R. China

## Abstract

Small RNAs, especially the microRNAs, have been revealed to play great roles in heart development and congenital heart defects. Several studies have shown dysregulated miRNAs in ventricular tissues of Tetralogy of Fallot (TOF) patients. In the present study, we conducted high throughput sequencing to obtain the global profiling of small RNA transcriptome in heart right ventricular samples from 10 age -matched TOF patients. These samples showed dominant composition of miRNA and mitochondrial associated RNAs. By sRNA cluster identification and differential gene expression analysis, significant sexual difference was discovered for sRNA expression in TOF patients. miR-1/miR-133, which have been identified as essential for cardiac development, account for the most variance of sRNA expression between sexes in TOF hearts.

## Introduction

During the normal process of heart development and growth, a genetic network involving precise temporal and spatial gene regulation is required. Abnormity in the network might lead to failure of cell lineage specification, pattern formation and cell migration necessary for cardiac development, which finally resulting in congenital heart defects (CHD). Most CHD cases are sporadic, which are most often inherited from unaffected parents. De novo mutation, incomplete penetrance and recessive mutation have been suggested to contribute to CHD inheritance. Whereas, only a small fraction of the cases could be genetically diagnosed.

Recently, small non-coding RNAs (sRNAs) have been shown to play important parts in cardiac gene expression network. microRNA (miRNA) with an average length of ~22 nucleotides is the most intensively investigated class of sRNAs, which tune gene expression through post-transcriptional regulation by means of reducing mRNA stability or inhibiting translation^1^. Heart development depends on proper spatio-temporal expression of particular miRNAs. For example, miR-1 is the first miRNA reported as essential for heart development^2^, which is cardiac specific. Members from miR-1 family and miR-133 family could function as pairs from the same bicistronic transcripts. miR-1 transgenic mics display similar phenotype as Hand2 mutant mice^2,3^; The miRNA-17–92 cluster have been suggested function in promoting cardiac progenitor differentiation in a dose-dependent manner; disruption of miR-138 by morpholino and antagomir in zebrafish could result in expression of atrioventricular canal specific genes in ventricular chamber, thus lead to abnormal cardiac patterning.

Tetralogy of Fallot (TOF) is the most common cyanotic CHD with an incidence of 10% in all CHD patients^4^. TOF is caused by non-uniform separation between truncus and bulbus arteriosus during early embryonic development, resulting in malformation such as ventricular septal defect, aortic overriding, pulmonary artery or right ventricular outflow tract stenosis, and right ventricular hypertrophy. Although the molecular mechanisms of isolated TOF still remains unclear. Several evidence from microarray analysis have shown that miRNAs should be dysregulated in TOF heart tissues^5–8^. These results emphasized the importance of miRNA in TOF etiology. However, a comprehensive profiling of sRNAs in TOF hearts have not been reported up to date. Furthermore, sexual difference in gene expression have been identified in left heart ventricle tissues with no prior cardiovascular disease using Genotype-Tissue Expression (GTEx) data^9^. In fact, in the rat model miR-1 have been discovered to be responsible for sexual difference of Cx43 expression in cardiomyocytes under pathologic conditions. we hypothesize that small RNA expression profile in male and female individuals with TOF should be different^10^.

In the present study, to get a full picture and characterize the sex differences of small RNA expression for TOF, we performed small RNA sequencing (small RNAseq) on 10 age- matched (5 female vs 5male) TOF right ventricular tissue samples. We found that TOF heart sRNA transcriptome have over represented composition of miRNAs and mitochondrion associated sRNAs, a significant difference of sRNA expression was also shown between male and female samples.

## Results

### Quality control of sequencing data

Sequencing data for each sample have a Q20 value greater than 97% and Q30 value greater than 95% (Table 1), suggesting good sequencing quality for all samples. After preprocessing, the proportion of clean reads retained ranged from 96.97–98.78% (Table 2).

**Table 1:** Sequencing reads statistics.

Sample	Reads	Bases(G)	Error rate (%)	Q20 (%)	Q30 (%)	GC content (%)
tof1	11592168	0.576	0.01	97.39	95.14	47.05
tof2	10268410	0.510	0.01	97.33	95.01	48.18
tof3	13857585	0.687	0.01	97.36	95.29	47.78
tof4	12425771	0.617	0.01	97.40	95.33	47.75
tof6	10044411	0.500	0.01	97.60	95.43	49.43
tof7	10652214	0.531	0.01	98.09	96.25	47.12
tof9	11078964	0.552	0.01	98.02	96.18	46.43
tof12	12276878	0.611	0.01	98.32	96.72	46.98
tof13	11089132	0.553	0.01	98.42	96.78	46.79
tof14	9954739	0.496	0.01	98.14	96.25	46.87

Note: we collected more samples and chose 10 with better RNA quality for RNAseq, thus the sample numbers are not consecutive.

**Table 2:** Quality control of reads preprocessing.

Sample	Total reads	N% > 10%	Low quality	5′adapter contamination	3′adapter null or insert null	With polly A/T/G/C	Clean reads
tof1	11592168 (100.00%)	2632 (0.02%)	66848 (0.58%)	157 (0.00%)	99645 (0.86%)	4770 (0.04%)	11418116 (95.80%)
tof2	10268410 (100.00%)	2265 (0.02%)	58783 (0.57%)	404 (0.00%)	96020 (0.94%)	6406 (0.06%)	10104532 (98.40%)
tof3	13857585 (100.00%)	1737 (0.01%)	108448 (0.78%)	206 (0.00%)	107758 (0.78%)	6225 (0.04%)	13633211 (98.38%)
tof4	12425771 (100.00%)	1468 (0.01%)	89623 (0.72%)	289 (0.00%)	111450 (0.90%)	5102 (0.04%)	12217839 (98.33%)
tof6	10044411 (100.00%)	1202 (0.01%)	44532 (0.44%)	292 (0.00%)	113812 (1.13%)	9080 (0.09%)	9875493 (98.32%)
tof7	10652214 (100.00%)	744 (0.01%)	33063 (0.31%)	117 (0.00%)	120102 (1.13%)	4662 (0.04%)	10493562 (98.51%)
tof9	11078964 (100.00%)	763 (0.01%)	41620 (0.38%)	114 (0.00%)	182186 (1.64%)	4761 (0.04%)	10849520 (97.93%)
tof12	12276878 (100.00%)	15 (0.00%)	48113 (0.39%)	263 (0.00%)	94668 (0.77%)	6124 (0.05%)	12127695 (98.78%)
tof13	11089132 (100.00%)	108 (0.00%)	26731 (0.24%)	88 (0.00%)	305766 (2.76%)	3839 (0.03%)	10752600 (96.97%)
tof14	9954739 (100.00%)	70 (0.00%)	24712 (0.25%)	71 (0.00%)	245656 (2.47%)	4004 (0.04%)	9680226 (97.24%)

### sRNA composition in TOF hearts

**Figure 1:**
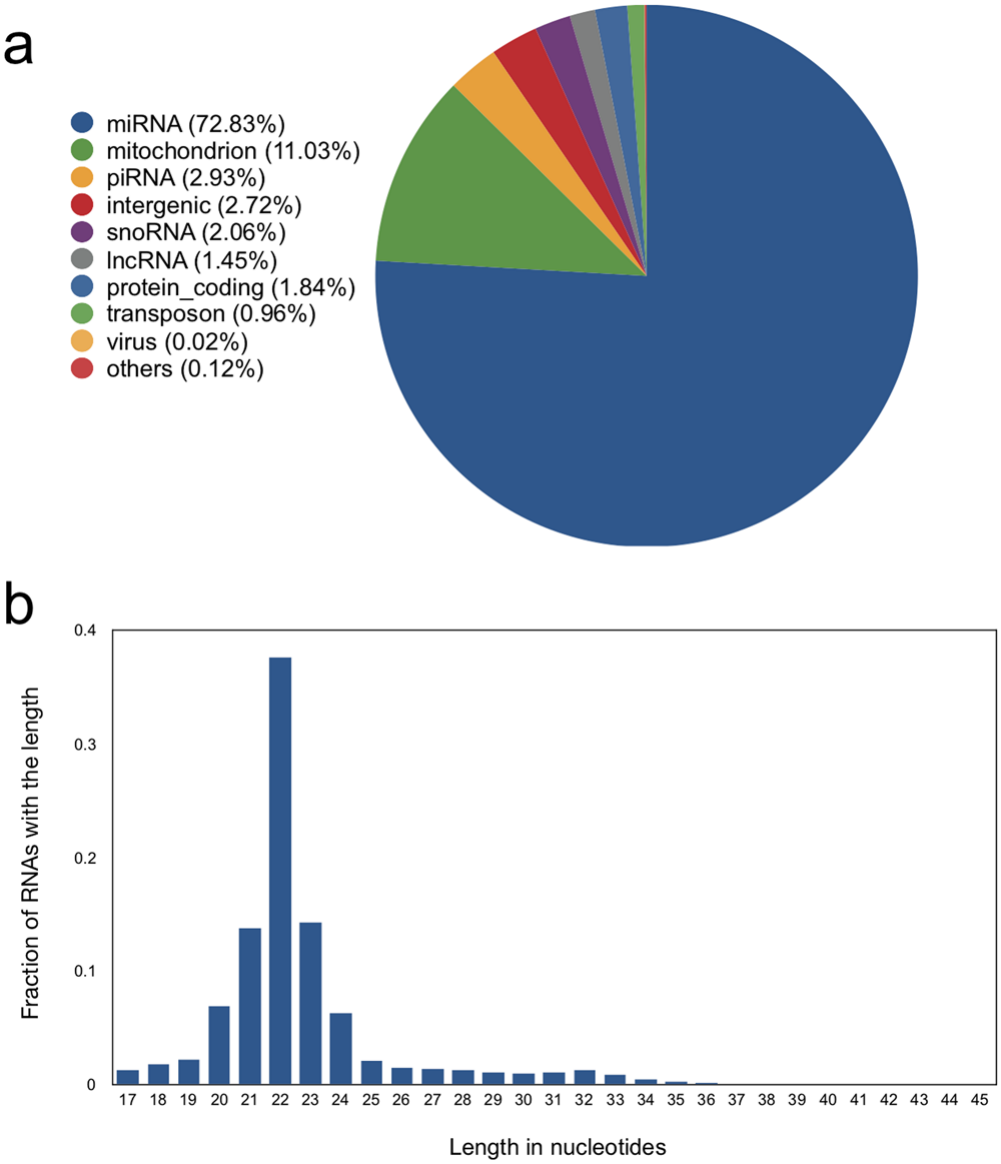
TOF right ventricular sRNA composition. (**a**) sRNA composition summed over all TOF samples. (**b**) Length distribution of sRNA summed over all TOF samples.

As expected, the most abundant class of sequenced sRNAs in TOF right ventricles is miRNA (72.83%). A substantial proportion of sRNA originated from mitochondrion sRNAs, which might be a unique feature for heart tissues since cardiomyocytes enormous mitochondrion. Other sRNA classes such as piRNA and snoRNA made relatively few contribution to the sRNA composition for TOF heart (Fig. 1a). In consistence with the dominant miRNA composition, the length distribution of sRNAs peaked at 22 bp (Fig. 1b).

### Detection of sRNA clusters in TOF hearts

A total of 600 sRNA clusters were identified using the SeqCluster analysis pipeline. Most of the clusters were mapped onto miRNA precursors (228, 38%). Additionally, 9.5% and 9.0% of the clusters mapped onto piRNAs and tRNAs (Supplementary Dataset 1).

### sRNA expression patterns separating male and female samples

**Figure 2:**
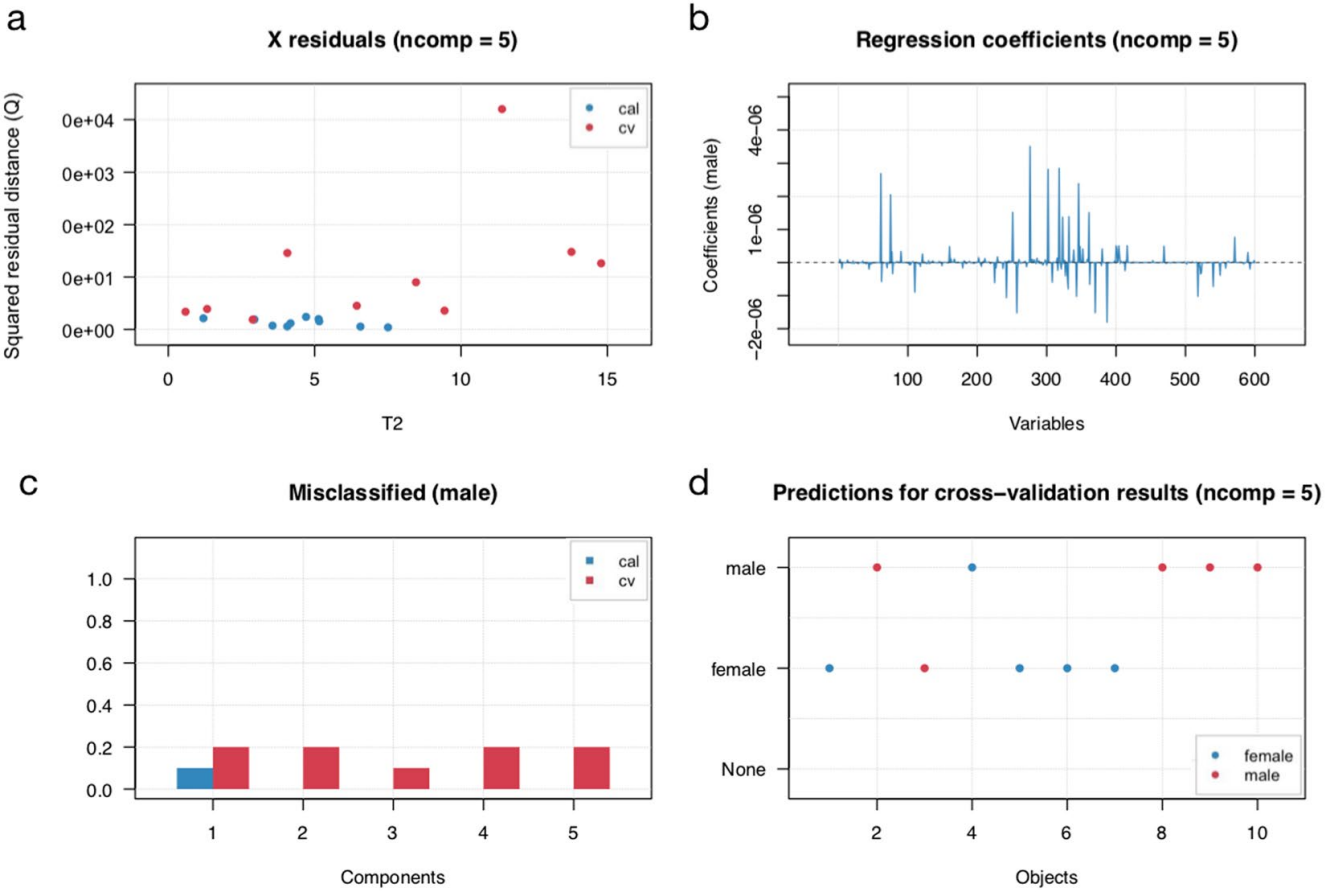
PLS-DA model statistics. (**a**) Plot with Q residulas vs Hotelling T2 values for PLS decomposition. (**b**) Regression coefficients of sRNA clusters. (**c**) Misclassified ratio plot. (**d**) Result of prediction for cross-validation results. ncomp, component used for model construction; cal, calibration; cv, cross validation.

**Figure 3:**
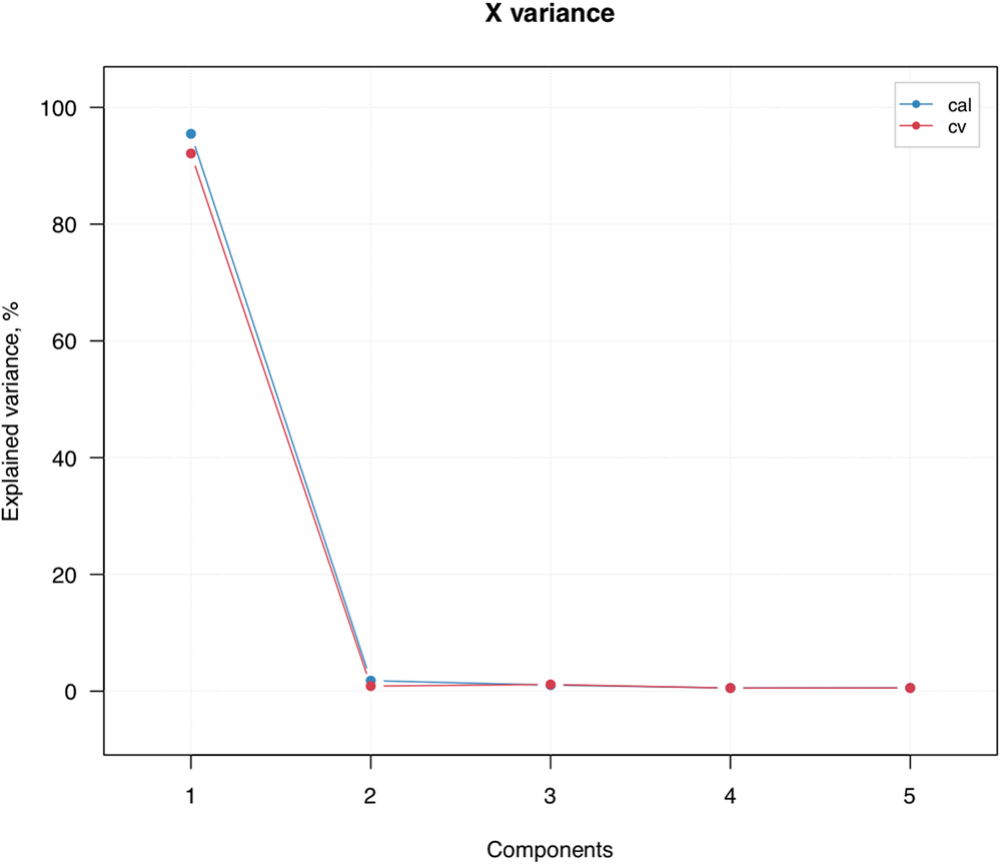
Plot with explained variance for cluster components.

PLS-DA was used to explore whether the sRNA expression pattern could distinguish between male and female samples. Using the sRNA cluster detected from SeqCluster a PLS-DA model (5 components were used) with specificity and sensitivity of 0.8 in cross validation (Fig. 2). The first component contributed to most of the performance (Fig. 3). The variable importance in projection (VIP) of PLS-DA is used to measure the relevance that each cluster contributed to the model. In our model, 24 cluster have a VIP score greater than 1.2. In addition, we also performed PLS-DA using miRNA identified with miraligner, but could not separate males and females (data not shown).

### Differential Expression of sRNAs between male and female TOF hearts

**Figure 4:**
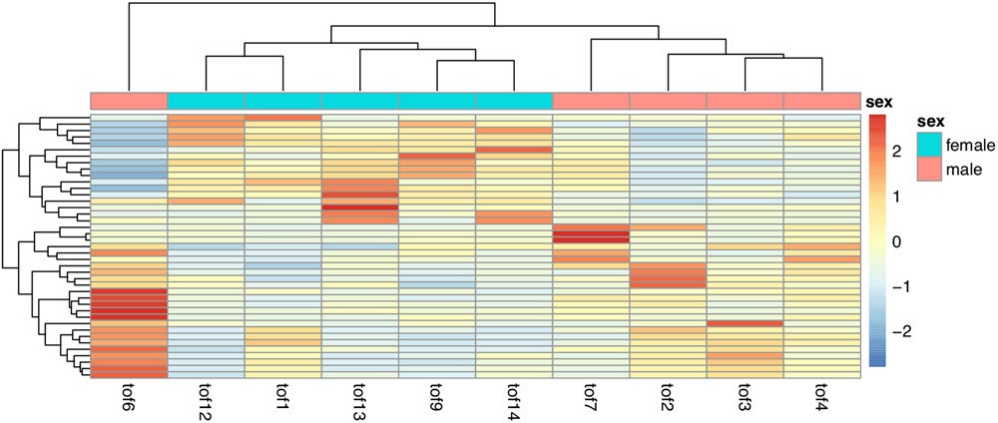
Heatmap of differentially expressed sRNA clusters among male and female TOF heart samples.

**Figure 5:**
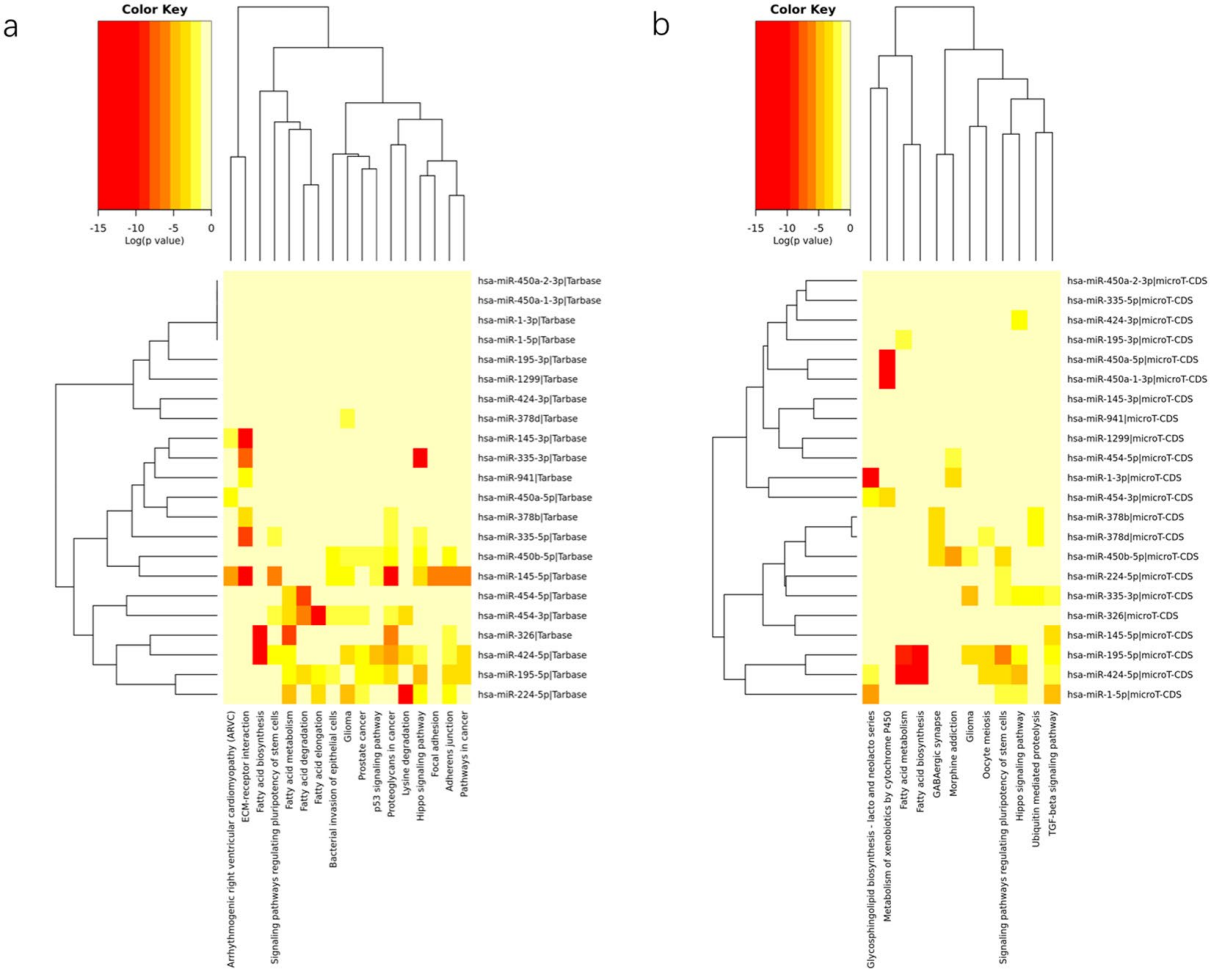
Heatmaps of KEGG pathway enrichment of sexually differential expressed sRNA clusters using (**a**) Tarbase and (**b**) microT-CDS data.

**Figure 6:**
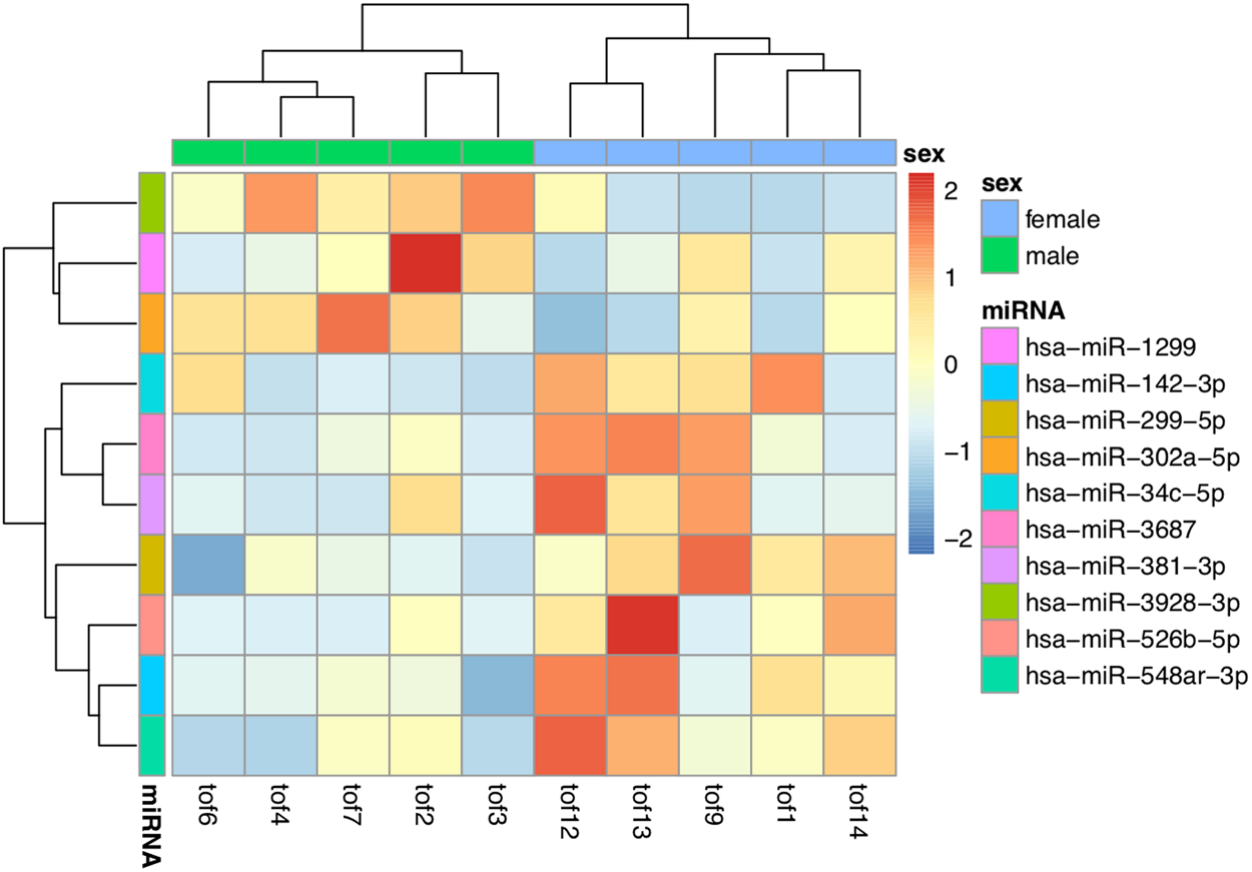
Heatmaps of differentially expressed miRNA identified by miraligner among male and female TOF heart samples.

Besides the identification of global patterns of sRNA clusters that could distinguish males from females. We also detected differential expression in single clusters between male and female TOF samples. Read counts were normalized (Supplementary Figure S1) and assessed. 41 significantly differently expressed sRNA clusters (nominal P value < 0.05) were identified (Fig. 4 and Supplementary Dataset 2). Targets of miRNAs in these clusters enriched in several KEGG pathways (Fig. 5). Differential expression were also performed for miRNAs annotated using miraligner. Totally 10 miRNAs were selected if miRNA isoforms (isomiRs) were not considered (Fig. 6). A corresponding list with isomiRs have been provided in Supplementary Dataset 3.

### Novel miRNA prediction

Using mirnovo, tens of novel miRNAs were predicted for the 10 TOF heart samples. The ranges of precision, sensitivity and specificity for the prediction model are 92.74~95.73%, 82.67~89.02, 96.39~98.60% respectively (Supplementary Figure S2). The list of novel miRNAs and their consensus sequences has been provided in Supplementary Dataset 4.

## Discussion

Our study provides the first systematic profiling of sRNA transcriptome in TOF, and the identification of sexually differentially expressed sRNAs. TOF right ventricles possess remarkable abundance of miRNAs, implying their importance in heart gene network regulation.

Using the read count information of the sRNA clustering we detected, a model with good performance on predicting sample sexes could be built. The first component could significantly explain more than 90% of the response variability. Surprisingly, the most important variable in the model is cluster 242 (VIP score: 3.08 × 10^2^) annotated with miR-1 and miR133 family. miR-1 have also been suggested might contribute to the development of TOF by regulating CX43 (connexin 43), which has been known to be involved in the development of conotruncal anomalies. Given the significant importance of this miRNA in heart development, we hypothesize that development of TOF in male and female patients have different molecular basis characterized by the expression of the miR-1/miR-133 cluster. It would be interesting to investigate that whether such difference exists in other types of CHD.

In our differential expression analysis, Clustering of the significant sRNA cluster (P value < 0.05) did not separate male and female samples perfectly, with tof1 located at the outgroup. We further use a larger (P value < 0.1) and smaller (P value < 0.03) gene set to repeat such clustering, females and males could be separated unambiguously in both result (Supplementary Figures S3 and S4). We finally retrieved the genes that confused the clustering of female samples, which showed inverse expression pattern as in other female samples (Supplementary Figure S5).

Among the differentially expressed miRNAs annotated using miraligner, 4 out of 10 was included in the 600 sRNA clusters. However, only hsa-miR-1299 corresponding to cluster 560 was detected as differentially expressed for sRNA clusters. Notably, KEGG pathway analysis indicated that targets of the sRNAs with sexually differential expression are enriched in several neurological pathways such as GABAergic synapse, Glioma. Regarding the fact that a large proportion of CHD cases have comorbidity of neurodevelopmental disorders, most of which also show great sex differences, It would be worth investigating the association of the differential expressed genes we identified with neurodevelopmental process.

In summary, our comprehensive analysis of sRNA transcriptome of TOF heart ventricular tissues revealed a specific pattern characterized by over represented miRNAs and mitochondrial related small RNAs. Significant difference was discovered when comparing the expression of sRNAs between male and female samples, miR-1/miR133 clusters should play a central role in the sexual difference. Furthermore, based on the sRNA sequence data, tens of novel miRNA sequences were predicted. Thus, our study provided a handful of candidate targets for revealing the molecular basis of TOF.

## Methods

### Ethical approval and informed consent

The Ethics Committee of the Shanghai Children’s Medical Center reviewed and approved this study (SCMCIRB-K2017009). All procedures performed in studies involving human participants were in accordance with the ethical standards of the institutional and/or national research committee and with the 1964 Helsinki declaration and its later amendments or comparable ethical standards. Informed consent was obtained from the patients’ parents (patients were under 18 years old).

### Subjects

Our subjects were patients with main cardiac malformation of TOF (Table 3) under the age of 1 year requiring surgical reconstruction. At surgery, diagnosis and anatomy of TOF were confirmed and entricular myocardial tissues were retrieved. Samples were immediately stored in RNALater (Ambion) at −80 °C for subsequent processing.

**Table 3:** Samples and the clinical phenotypes.

Sample	Age	Sex	Phenotype
tof1	6 month	female	TOF/PFO
tof2	6 month	male	TOF/PDA
tof3	1 year 2 month	male	TOF
tof4	6 month	male	TOF
tof6	8 month	male	TOF
tof7	6 month	male	TOF
tof9	6 month	female	TOF/ASD/PDA
tof12	4 month	female	TOF/ASD
tof13	8 month	female	TOF
tof14	6 month	female	TOF

TOF: Tetralogy of Fallot; PFO: Patent Foramen Oval; PDA: Patent Ductus Arteriosus; ASD: Atrial Septal Defect.

### RNA extraction, quantification and qualification

Tissue samples preserved in RNALater was lysed in Trizol (Ambion). Total RNA was extracted using the miRNeasy kit (Qiagen, Hilden, Germany) according to the manufacturer’s instructions. The RNA degration and contamination was monitored by 1% agarose gels electrophoresis. The concentration of RNA samples were measured using Qubit RNA Assay Kit in Qubit 2.0 Flurometer (Life Technologies, CA, USA). The RNA purity was checked using the NanoPhotometer spectrophotometer(IMPlLEN, CA, USA). The RNA integrity was assessed using the RNA Nano 6000 Assay Kit of the 2100 Bioanalyzer instrument (Agilent Technologies, Palo Alto, CA).

### Small RNA library construction and sequencing

A total amount of 3 μg total RNA for each sample was used for small RNA library construction. Sequencing libraries were generated using NEBNext Multiplex Small RNA Library Prep Set for Illumina (NEB, Beverly, MA, USA.) following manufacturer’s recommendations and index codes were ligated to attribute sequences to each sample. Briefly, NEB 3′ SR Adaptor was specifically added to 3′ end of miRNA, siRNA and piRNA. The SR RT Primer hybridized to the excess of 3′ SR Adaptor and transformed the single-stranded DNA adaptor into a double-stranded DNA molecular after 3′ ligation reaction. The first strand cDNA was then synthesized using M-MuLV Reverse Transcriptase (RNase H-). PCR amplification was performed using LongAmp Taq 2X Master Mix, SR primer for illumine and index primer. The PCR products were purified on an 8% polyacrylamide gel (100 V, 80 min). DNA fragments corresponding to 140~160 bp were recovered and dissolved in 83 μL elution buffer. At last, library quality was determined on the Agilent Bioanalyzer 2100 system using DNA High Sensitivity Chips.

The clustering of index-coded samples was performed on a cBot Cluster Generation System using TruSeq SR Cluster Kit v3-cBot-HS(Illumina) according to the manufacturers’ instructions. The library preparations were then sequenced on an Illumina Hiseq. 2500/2000 platform and 50 bp single-end reads were generated.

### Raw sequencing reads preprocessing

For raw reads of each sample, adapter sequences were trimmed cutadapt^11^. Reads with low quality (e.g. proportion of bases with sQ < = 5 greater than 50%; proportion of N greater >10%; 5′adaptor contamination) were also removed.

### Genome mapping, annotation and sRNA cluster detection

sRNA clusters were detected using the SeqCluster pipeline^12–17^. Sequences with adapter removal were then collapsed among samples, generating a set of unique reads with the corresponding counts. Sequences with more than 10 counts were used for further analysis. The reads were mapped onto the human genome (hg38) using STAR^18,19^ implementing 2-pass alignment mode. Clusters were then detected. Annotations used were GENCODE27 (GRCh38.p10), transposon annotations from Repbase^20^. snoRNA and miRNA annotations from the UCSC table browser^21^. In SeqCluster, a group of at least 10 overlapping sequences mapping onto a specific genomic site was defined as a hotspot and a cluster was defined as hotspots sharing any number of sequences. A cluster represents a group of a type of sRNAs that are consistently co-expressed.

In addition, we used miraligner for miRNA alignment and annotation using the miRBase dataset for each sample.

### Partial least squares discriminant analysis

Partial least squares discriminant analysis (PLS-DA) is a method appropriate for dimension reduction for datasets with multicollineality and was successfully used in sRNA analysis^22^. We implemented this method in sRNA cluster and miRNAs we identified for the TOF samples using the R package mdatools.

### Differential expression analysis

We used the R package DESeq2^23^ for differential expression analysis between male and female samples. The analyses were conducted for read counts generated from SeqCluster and miraligner respectively. Clustering of gene sets among samples were visualized using the R package pheatmap (Euclidian distance, wald method).

### Pathway enrichment analysis of miRNA targets

Differential expressed miRNAs were submitted to web based DIANA TOOLs miPath v3.0 for KEGG pathway enrichment analysis using the default parameters. Tarbase v7.0 and microT-CDS datasets were used as target information for miRNAs. Pathway union were used to generate pathway clustering heatmaps.

### Novo miRNA prediction

A machine learning based tool-mirnovo, was used to predict the novel miRNA sequences from our sRNA data^24^.

## Electronic supplementary material

Supplementary Figures

Dataset 1

Dataset 2

Dataset 3

Dataset 4

## Data Availability

RNA-Seq data has been submitted to the NCBI Gene Expression Omnibus (GSE113511).
