# Supplementary material for: Sexual difference of small RNA expression in Tetralogy of Fallot
Source: Sci Rep. 2018 Aug 27;8:12847. doi: 10.1038/s41598-018-31243-6 (PMC6110777; doi:10.1038/s41598-018-31243-6)
Supplement: Supplementary file 1 — Supplementary Figures [file 41598_2018_31243_MOESM1_ESM.pdf]

Sexual difference of small RNA expression in Tetralogy of Fallot

Bo Wang[1] Guocheng Shi[2] Zhongqun Zhu[2] Huiwen Chen[2*] Qihua Fu[1*]

1Department of Laboratory Medicine, Shanghai Children’s Medical Center, Shanghai Jiao Tong University School of Medicine, Shanghai, 200127, P. R. China

2 Department of Cardiothoracic Surgery, Heart Center, Shanghai Children's Medical Center, Shanghai Jiaotong University School of Medicine, Shanghai, 200127, P. R. China

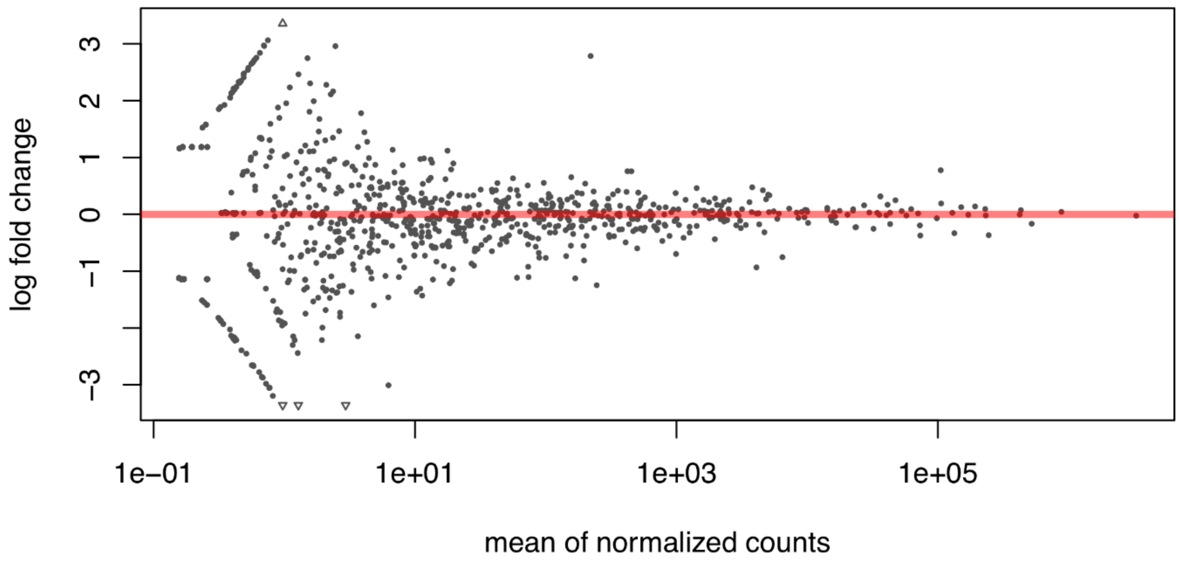

Figure S1. Mean value of normalized read counts.

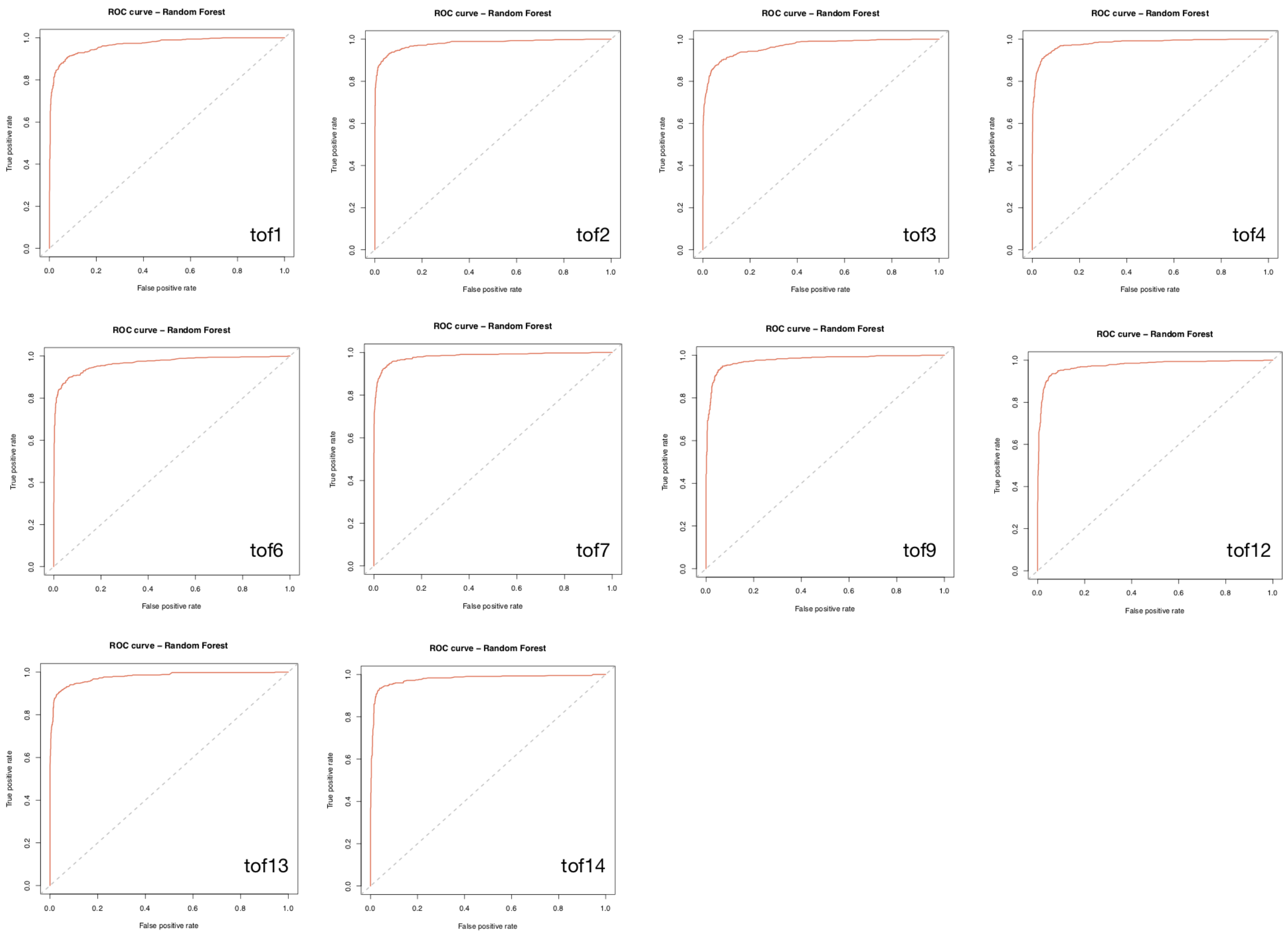

Figure S2. ROC curves for novel miRNA prediction.

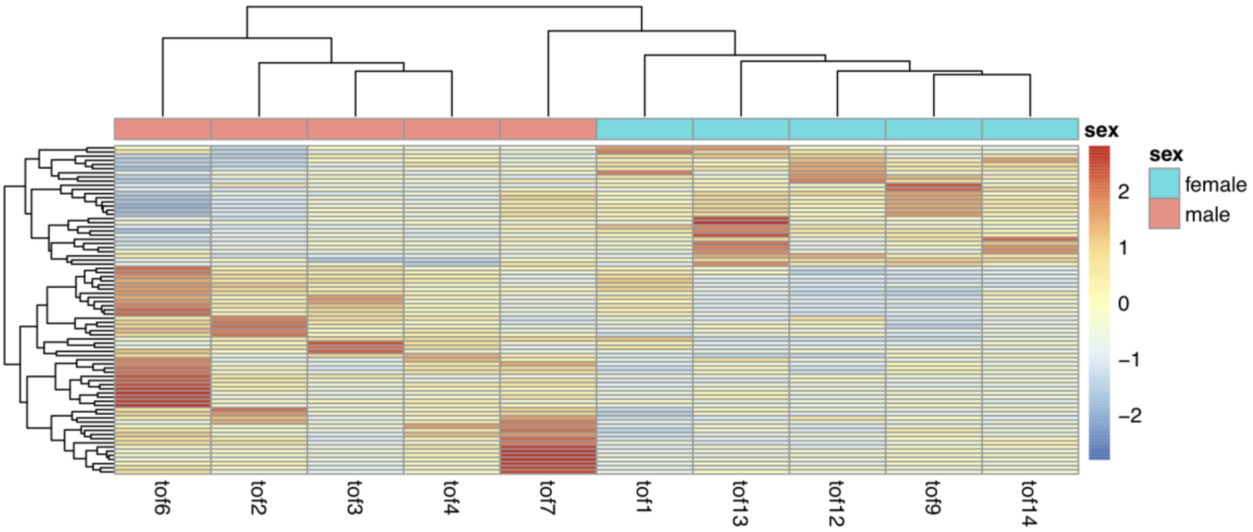

Figure S3. Clustering using sRNA clusters with sexual difference (P<0.1).

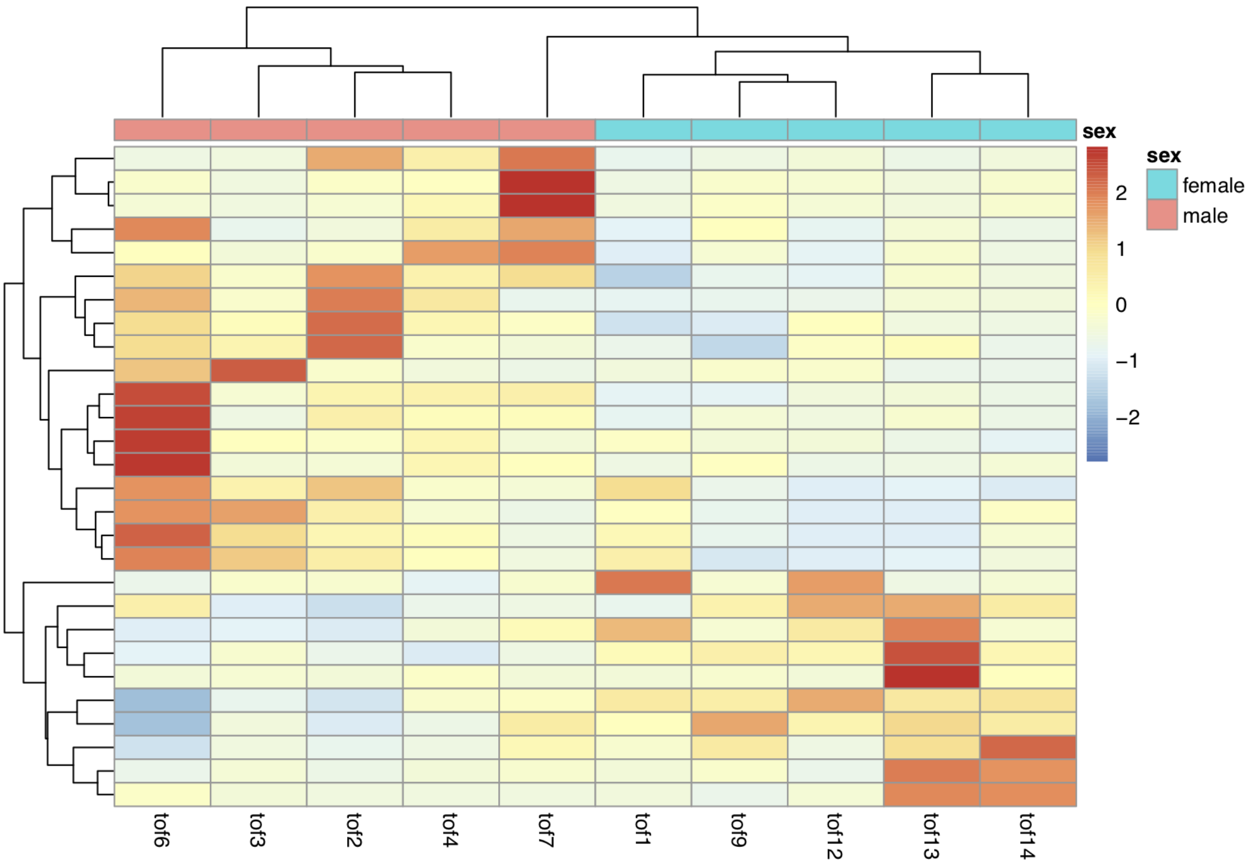

Figure S4. Clustering using sRNA clusters with sexual difference (P<0.03).

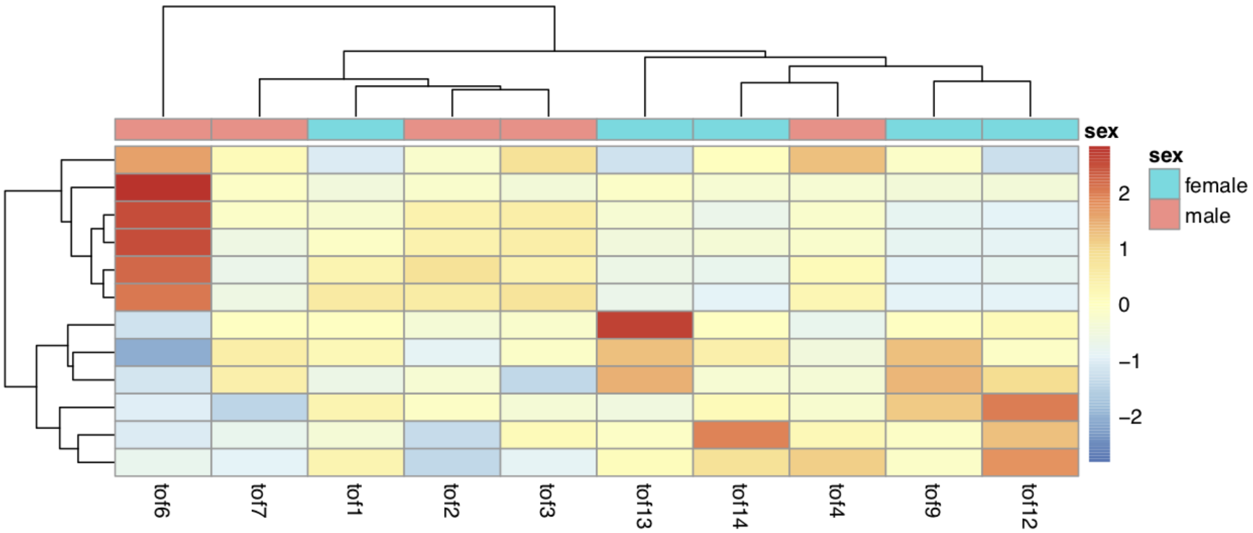

Figure S5. Genes that confused the clustering of female sample.
